# Immune infiltration landscape on prognosis and therapeutic response and relevant epigenetic and transcriptomic mechanisms in lung adenocarcinoma

**DOI:** 10.3389/fimmu.2022.983570

**Published:** 2022-10-06

**Authors:** Liangming Zhang, Biwang Jiang, Zhuxiang Lan, Chaomian Yang, Yien Yao, Jie Lin, Qiu Wei

**Affiliations:** ^1^ Department of Pulmonary and Critical Care Medicine, The Fifth Affiliated Hospital of Guangxi Medical University, Nanning, China; ^2^ Department of Pulmonary and Critical Care Medicine, The First People’s Hospital of Nanning, Nanning, China

**Keywords:** lung adenocarcinoma, prognosis, immune cells, immunity, therapeutic response, methylation, EXO1

## Abstract

**Objective:**

Lung adenocarcinoma (LUAD) is the most prevalent lung cancer subtype, but its immune infiltration features are not comprehensively understood. To address the issue, the present study was initiated to describe the immune infiltrations across LUAD from cellular compositional, functional, and mechanism perspectives.

**Methods:**

We adopted five LUAD datasets (GSE32863, GSE43458, GSE75037, TCGA-LUAD, and GSE72094). Differentially expressed genes between LUAD and controls were selected for co-expression network analysis. Risky immune cell types were determined for classifying LUAD patients as diverse subtypes, followed by a comparison of antitumor immunity and therapeutic response between subtypes. Then, LUAD- and subtype-related key module genes affected by DNA methylation were determined for quantifying a scoring scheme. EXO1 was chosen for functional analysis *via in vitro* assays.

**Results:**

Two immune cell infiltration-based subtypes (C1 and C2) were established across LUAD, with poorer prognostic outcomes and lower infiltration of immune cell types in C1. Additionally, C1 presented higher responses to immune checkpoint blockade and targeted agents (JNK inhibitor VIII, BI-D1870, RO-3306, etc.). The scoring system (comprising GAPDH, EXO1, FYN, CFTR, and KLF4) possessed higher accuracy in estimating patients’ prognostic outcomes. EXO1 upregulation contributed to the growth, migration, and invasion of LUAD cells. In addition, EXO1 facilitated PD-L1 and sPD-L1 expression in LUAD cells.

**Conclusion:**

Altogether, our findings offer a comprehensive understanding of the immune infiltration landscape on prognosis and therapeutic response of LUAD as well as unveil potential epigenetic and transcriptomic mechanisms, which might assist personalized treatment.

## Introduction

Lung adenocarcinoma (LUAD) is the most prevalent lung cancer subtype, with rising frequency (1). Despite the long-term exposure to tobacco smoke as the most frequent etiology, it affects 15%–20% of cases in non-smokers and is usually attributed to integrated genetic and environmental factors (2). This disease is characterized by extensive heterogeneity considering its clinical behavior and molecular landscape (3). Although early detection and personalized medicine have been improved, a sizable fraction of patients in the early stages would experience relapse and adverse clinical outcomes (4). Hence, a potent therapeutic schedule is required for LUAD treatment.

Immune checkpoint blockade (ICB) of PD1/PD-L1 may unleash CD8+ T cells and exert a potent antitumor response, which has gained approval for non-small cell lung cancer therapy (5). The clinical efficacy greatly depends upon individual tumor microenvironment (TME) and immunity-relevant regulatory network (6), underscoring the significance of immune infiltrates in orchestrating TME as well as affecting patients’ survival (7). Regrettably, only 20% of cases benefit from ICB, an alarmingly low number under high mutational burden as well as immune infiltrations investigated in lung cancer (8). Considerable effort has been devoted to developing precise predictive immuno-oncology biomarkers (tumor mutational burden (TMB), PD-L1 expression, etc.) (9). Nonetheless, currently, there are still no uniform criteria or single indicators for selecting LUAD patients to receive ICB, thus drawing attention to comprehending the mechanisms underlying antitumor immunity in LUAD. The current study reported a comprehensive analysis of the immune cell infiltration landscape *via* the integration of epigenetic and transcriptomic profiles of LUAD from public databases. Here, we delineated two immune cell infiltration subtypes across LUAD and dissected relevant epigenetic and transcriptomic mechanisms. In addition, a relevant scoring scheme was built to infer individual LUAD patients’ prognoses. *In vitro* assays demonstrated the function of EXO1 (a key gene) in the malignant progression of LUAD.

## Materials and methods

### Data acquisition and preprocessing

Four datasets—GSE32863 (https://www.ncbi.nlm.nih.gov/geo/query/acc.cgi?acc=GSE32863) (10), GSE43458 (https://www.ncbi.nlm.nih.gov/geo/query/acc.cgi?acc=GSE43458) (11), GSE75037 (https://www.ncbi.nlm.nih.gov/geo/query/acc.cgi?acc=GSE75037) (12), and GSE72094 (https://www.ncbi.nlm.nih.gov/geo/query/acc.cgi?acc=GSE72094)—were acquired from the Gene Expression Omnibus (GEO) repository. The GSE32863 dataset comprised microarray expression profiling of 58 pairs of LUAD and adjacent non-tumor lung tissues on the Illumina platform, which were log2-transformed and subjected to robust spline normalization with the lumi package (13). Gene expression profiles in the GSE43458 (containing 80 LUAD and 30 normal lung tissues) and GSE72094 (containing 442 LUAD specimens) datasets on the Affymetrix platform were normalized, summarized, and adjusted for background with robust multichip average, together with log2 transformation utilizing integrated BRB-Array Tools. The GSE75037 dataset included expression profiling of 83 pairs of LUAD and matched adjacent non-malignant lung tissues on the Illumina platform. Bead-level data were background-adjusted and summarized with model-based background correction for the BeadArrays algorithm (14), followed by quantile normalization and log2 transformation. Moreover, RNA sequencing data of 510 primary LUAD and 58 normal tissues were obtained from The Cancer Genome Atlas (TCGA; https://portal.gdc.cancer.gov/), and count data were normalized with Trimmed Mean of M-values utilizing the edgeR package (15). Gene methylation data of 473 LUAD and 34 normal tissues were also downloaded from TCGA on the basis of the Infinium HumanMethylation450 BeadChip array.

### Differentially expressed gene identification

The expression levels of genes between LUAD and normal lung tissues in each dataset (GSE32863, GSE43458, GSE75037, and TCGA-LUAD) were compared with Student’s t-test utilizing the limma package. The p-value was adjusted through the false discovery rate. Differentially expressed genes (DEGs) were identified in accordance with adjusted p < 0.05 (16).

### Weighted gene correlation network analysis

The co-expression network of DEGs shared by four datasets was constructed *via* the weighted gene correlation network analysis (WGCNA) package (17). The soft-thresholding power β of 4 (scale-free R^2^ of 0.9) was utilized for achieving an adjacency matrix with scale-free topology. Then, the topological overlap matrix was established for determining the connectivity and dissimilarity of the co-expression network. With the use of the dynamic tree cut approach, co-expression modules were clustered. Pearson’s test was utilized for computing the relationships between module eigengene (ME), which is defined as the first principal component of a given module and clinical traits.

### Immune infiltrate estimation

Based on the markers of immune infiltrates, the enrichment score computed by single-sample gene set enrichment analysis (ssGSEA) was utilized for denoting the relative infiltration of each immune cell type (18). Univariate Cox regression analysis was implemented for inferring the relationships between immune infiltrates with overall survival (OS).

### Clustering analysis

In accordance with the infiltration levels of risky immune cell types, a hierarchical clustering dendrogram was adopted for classifying LUAD cases as the optimal number of clusters in TCGA-LUAD and GSE72094 datasets. The Elbow method was used to validate the number of clusters. OS between clusters was evaluated *via* Kaplan–Meier approach together with the log-rank test.

### Functional enrichment analysis

Functional enrichment analysis of module genes in certain Gene Ontology (GO) terms and Kyoto Encyclopedia of Genes and Genomes (KEGG) pathways was evaluated with the clusterProfiler package (19). GO terms comprised biological processes (BPs) and cellular components (CCs) together with molecular functions (MFs). Gene set enrichment analysis (GSEA) software was adopted for determining gene sets with significant differences between groups (20). The pathway activity in each sample was estimated utilizing gene set variation analysis (GSVA) in accordance with the enrichment of gene sets (18). Terms with adjusted p < 0.05 were considered significant enrichment.

### Drug sensitivity estimation

The IC50 value of therapeutic agents was estimated with the pRRophetic approach (21), which can denote the effect of an agent in mitigating specific biological or biochemical functions.

### Identification of CpG sites in lung adenocarcinoma

Differentially methylated sites were determined with the ChAMP package (22). First, quality control was implemented with the champ.QC function. Second, the β value was normalized *via* champ.norm function. Differentially methylated genes were screened according to the CpG site with p ≤ 0.05.

### Mutational analysis

TMB denotes the number of somatic mutations in each megabase of the query genome sequence (23), which was computed from the number of variants out of the total length of the human exons (38 million). A waterfall diagram was conducted with the maftools package for assessing the number of somatic mutations across TCGA-LUAD specimens (24).

### Protein–protein interaction

Module genes with methylation were imported into the online tool STRING (https://string-db.org) (25). The protein–protein interaction (PPI) network with a combined score of ≥0.7 (high confidence) was constructed and visualized with Cytoscape software (26). Genes showing more interactions with others were defined as key module genes.

### Nomogram construction

Key module genes were utilized to establish a multivariate Cox regression model with a survival package, and the risk score was computed by combining the coefficient and mRNA level of each key module gene, following the formula: risk score = β_1_x_1_ + β_2_x_2_ + ··· + β_i_x_i_ (where β_i_ represents the coefficient of gene i and x_i_ represents the mRNA level of gene i). With the median value, LUAD cases were classified as high- and low-risk groups. Survival state was compared between groups. Afterward, a key module gene-based nomogram was developed *via* the rms package for estimation of 5- and 8-year OS. The prediction accuracy of the nomogram was assessed *via* calibration curves.

### Cell culture and transfection

Human lung normal epithelial cell line (BEAS-2B) and LUAD cell lines (NCI-H1975, NCI-H1395, A549, and PC9) were cultivated in Roswell Park Memorial Institute (RPMI) 1640 medium (Thermo Scientific, MA, USA) plus 10% fetal bovine serum (FBS) at 37°C in a humidified incubator with an atmosphere of 5% CO_2_. The coding sequence of full-length human EXO1 was subcloned into pLenti-CMV-GFP-Puro (#17448; Addgene, MA, USA) following the manufacturer’s instructions. A549 cells were treated with pLenti-CMV-GFP-Puro-EXO1 or matched empty vector as negative control (NC) under 8 µg/ml of polybrene for 12 h. NCI-H1975 cells were transfected with 10 nmol/L short interfering RNAs (siRNAs) against EXO1 (siRNAs 1-5, Hanyin, Shanghai, China) or a scrambled negative control siRNA (siRNA NC; Hanyin) *via* Lipofectamine 2000 (Invitrogen, MA, USA) in line with the manufacturer’s instructions. The sequences included the following: EXO1 siRNA1, 5′-GAAGAGAAGTTTCGTTACA-3′; EXO1 siRNA2, 5′-GTTGGCCTATCTTAACAAA-3′; EXO1 siRNA3, 5′-GAAGTAGAGAGATCTAGAA-3′; EXO1 siRNA4, 5′-TGACTACAATCCAGACACT-3′; EXO1 siRNA5, 5′-CCATTTCACCACCCACTTT-3′. Quantitative real-time PCR was adopted to examine EXO1 expression after transfection.

### Quantitative real-time PCR

Total RNA was isolated with TRIzol reagent (Thermo Scientific) following the manufacturer’s instructions and reverse-transcribed into cDNA with a reverse transcription kit (Takara, Dalian, China). Quantitative PCR was conducted with SYBR Green PCR Mix (Invitrogen, MA, USA) following the primers: EXO1 forward, 5′-TGAGGAAGTATAAAGGGCAGGT-3′; EXO1 reverse, 5′-AGTTTTTCAGCACAAGCAATAGC-3′; GAPDH forward, 5′-GGAGCCAAAAGGGTCATCATCTC-3′; GAPDH reverse, 5′-GAGGGGCCATCCACAGTCTTCT-3′; PD-L1 forward, 5′-TGGCATTTGCTGAACGCATTT-3′; PD-L1 reverse, 5′-TGCAGCCAGGTCTAATTGTTTT-3′. EXO1 mRNA level was computed with the 2^−ΔΔCt^ approach and normalized to GAPDH.

### Cellular proliferation assay

Transfected cells were seeded into 96-well plates (1 × 10^3^ cells/well) and cultivated for 5 days. Cellular proliferation was measured at 0, 1, 2, 3, and 4 days with Cell Counting Kit-8 (CCK-8; Beyotime, Shanghai, China). Cultures were exposed to CCK-8 reagent for 2 h. Afterward, the optical density (OD) value at 450 nm was measured.

### Transwell assay

Migration and invasion were examined utilizing transwell chambers (Corning, MA, USA). For migration assay, transfected cells were cultivated in serum-free RPMI 1640 medium for 15 h and then loaded into the upper chamber. The lower chamber was filled with RPMI 1640 medium with 20% FBS. For invasion assay, the upper chamber was coated with 25 µg of Matrigel (BD, CA, USA) and then seeded with 5 × 10^4^ cells. RPMI 1640 medium was added to the lower chamber. Following 24-h incubation, migratory and invasive cells were fixed with glutaraldehyde and dyed with crystal violet.

### Western blot

The cells were lysed in radioimmunoprecipitation assay (RIPA) buffer (Beyotime, China) plus a protease inhibitor cocktail, followed by protein quantification utilizing the bicinchoninic acid (BCA) method. The lysates were subjected to sodium dodecyl sulfate–polyacrylamide gel electrophoresis (SDS-PAGE) and transferred onto the polyvinylidene difluoride (PVDF) membrane. The membrane was immersed in 5% skim milk and probed with PD-L1 (1/1000; ab213480; Abcam, Waltham, MA, USA) and GAPDH (1/2500; ab9485; Abcam) overnight at 4°C. Afterward, a secondary antibody (1/5000; ab7097; Abcam) was applied to the membrane. Protein bands were visualized utilizing an enhanced chemiluminescence (ECL) substrate (Solarbio, Beijing, China).

### Enzyme-linked immunosorbent assay

In accordance with the manufacturer’s specification, soluble PD-L1 (sPD-L1) level was detected utilizing a human PD-L1 ELISA kit (ED-16408; Xiamen Lunchangshuo Biotechnology Co., Ltd., China).

### Statistical analysis

Data were expressed as mean ± standard deviation. Statistical analysis was conducted with GraphPad Prism 8.0.1 (GraphPad Software, CA, USA) or R language 4.0.5. The difference was evaluated for significance with Student’s t-test, Wilcoxon rank-sum test, and one-way or two-way analysis of variance. Pearson’s or Spearman’s test was utilized for correlation analysis. Receiver operating characteristic (ROC) curve of each key module gene was drawn, and the area under the curve (AUC) was computed for estimating the prediction accuracy utilizing the survivalROC package. p < 0.05 denoted statistical significance.

## Results

### Identification of differentially expressed genes in lung adenocarcinoma and their co-expression modules

**Figure 1:**
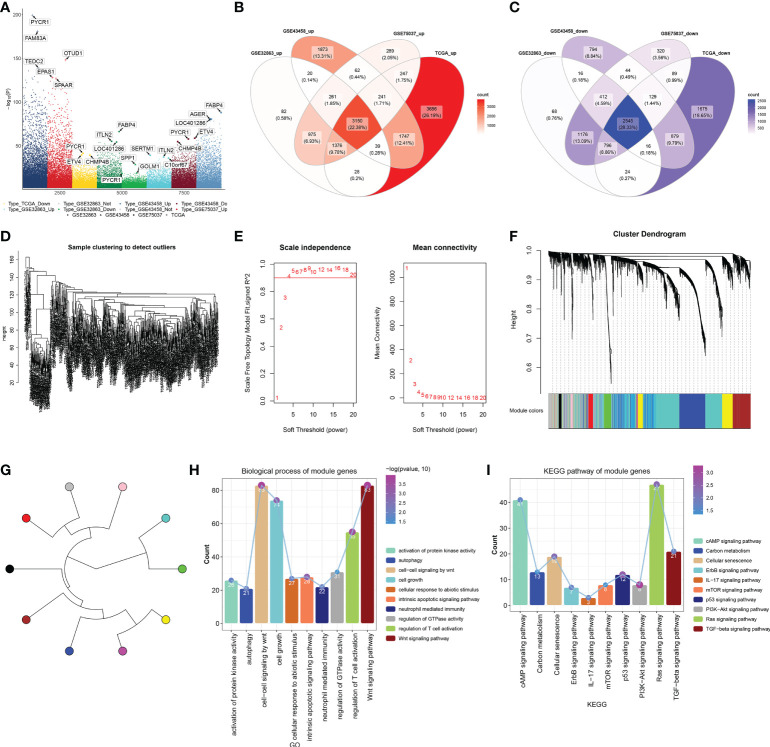
Identification of DEGs in LUAD and their co-expression modules. **(A)** Genes with notable upregulation or downregulation in LUAD than controls in four LUAD datasets (GSE32863, GSE43458, GSE75037, and TCGA-LUAD). **(B, C)** Venn diagrams of upregulated or downregulated genes shared by above four datasets. **(D)** Sample clustering to detect outliers. **(E)** Calculation of scale independence or mean connectivity under diverse soft‐thresholding power β values. **(F)** Co-expression modules based on DEGs shared by above four datasets with average linkage clustering. **(G)** Co-expression module clustering diagram. **(H, I)** Biological processes and KEGG pathways of module genes. DEGs, differentially expressed genes; LUAD, lung adenocarcinoma; KEGG, Kyoto Encyclopedia of Genes and Genomes.

The current study retrospectively collected four LUAD datasets (GSE32863, GSE43458, GSE75037, and TCGA-LUAD). A total of 16,667 DEGs were selected by comparing LUAD with controls in four datasets (**Figure 1A**). Among them, 3,150 upregulated genes and 2,545 downregulated genes were shared by four datasets (**Figures 1B, C**). After detection of outliers (**Figure 1D**), the WGCNA approach was adopted to determine different modules for shared DEGs in accordance with scale-free fit index β = 4 (scale-free R^2^ = 0.9; **Figure 1E**). Average linkage clustering generated nine co-expression modules containing 4,849 shared DEGs (**Figures 1F, G**). Module genes were notably in relation to immunity (neutrophil-mediated immunity, regulation of T-cell activation, IL-17 signaling pathway, etc.) and tumorigenic (autophagy, Wnt/cAMP/ErbB/mTOR/p53/PI3K-Akt/Ras/TGF-beta signaling pathways, etc.) processes and pathways (**Figures 1H, I**). The detailed information is listed in **Supplementary Table 1**.

### Definition of immune cell infiltration-based classification across lung adenocarcinoma

**Figure 2:**
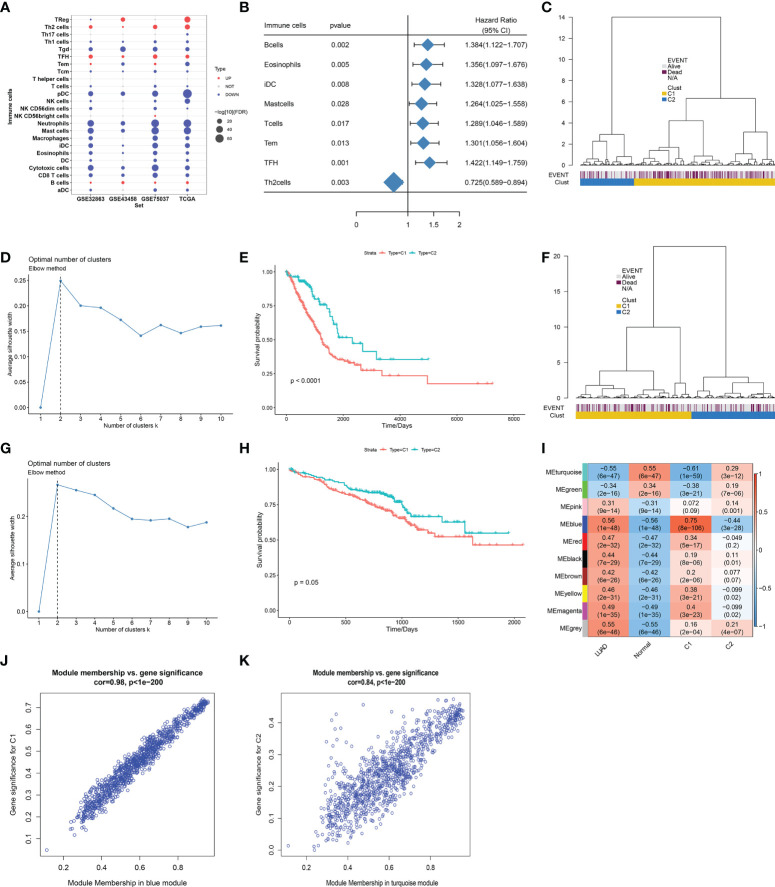
Definition of immune cell infiltration-based classification across LUAD. **(A)** Differences in infiltration levels of immune cell types between LUAD and normal lung tissues in four datasets (GSE32863, GSE43458, GSE75037, and TCGA-LUAD). **(B)** Forest plot of immune cell types whose infiltration levels significantly correlated to TCGA-LUAD cases’ OS. **(C)** Definition of two molecular subtypes across TCGA-LUAD on the basis of infiltration levels of seven risky immune cell types. **(D)** Elbow method for validating the accuracy of this molecular classification across TCGA-LUAD samples. **(E)** Kaplan–Meier curves of OS between two subtypes in TCGA-LUAD cohort. **(F)** Validation of this immune cell infiltration-based classification in the GSE72094 cohort. **(G)** Elbow method for validating the optimal number of clusters in the GSE72094 cohort. **(H)** Kaplan–Meier curves of OS between two subtypes in the GSE72094 cohort. **(I)** Heatmap of the associations between co-expression modules and clinical traits (LUAD and normal tissues; C1 and C2 subtypes). **(J)** Scatter plots of the correlation between module membership in MEblue module and gene significance for C1 subtype. **(K)** The correlation between module membership in MEturquoise module and gene significance for C2 subtype. LUAD, lung adenocarcinoma; OS, overall survival.

Infiltration levels of immune cell types notably differed between LUAD and control lung tissues. B cells, follicular helper T (Tfh) cells, regulatory T cells (Tregs), and T helper 2 (Th2) cells were abundant in LUAD, with other immune cell types (neutrophils, mast cells, etc.) abundant in normal lungs (**Figure 2A**). Eight immune cell types prominently correlated to LUAD cases’ OS, of which B cells, eosinophils, immature dendritic cells (iDCs), mast cells, T cells, effective memory T (Tem), and Tfh cells acted as risk factors of OS, with Th2 cells as a protective factor (**Figure 2B**). On the basis of infiltration of the above seven risky immune cell types, we classified LUAD patients in TCGA-LUAD cohort as two molecular subtypes, namely, C1 and C2 (**Figure 2C**). The Elbow method confirmed the accuracy of this molecular classification (**Figure 2D**). In **Figure 2E**, OS outcomes observably differed between subtypes, with the survival advantage in C2. In addition, we externally verified this immune cell infiltration-based classification in the GSE72094 dataset. As expected, similar results were observed, as shown in **Figures 2F–H**. Further analysis found that the MEblue module exhibited the strongest association with LUAD and C1 subtype, with the strongest correlation between the MEturquoise module and normal lung tissues and the C2 subtype (**Figure 2I**). For DEGs in the MEblue module, module membership dramatically correlated to gene significance for the C1 subtype (**Figure 2J**). Moreover, for DEGs in the MEturquoise module, module membership was markedly associated with gene significance for the C2 subtype (**Figure 2K**). Thus, MEturquoise and MEblue were regarded as the key modules.

### Signaling pathways, antitumor immunity, and drug sensitivity in two immune infiltrate-based subtypes

**Figure 3:**
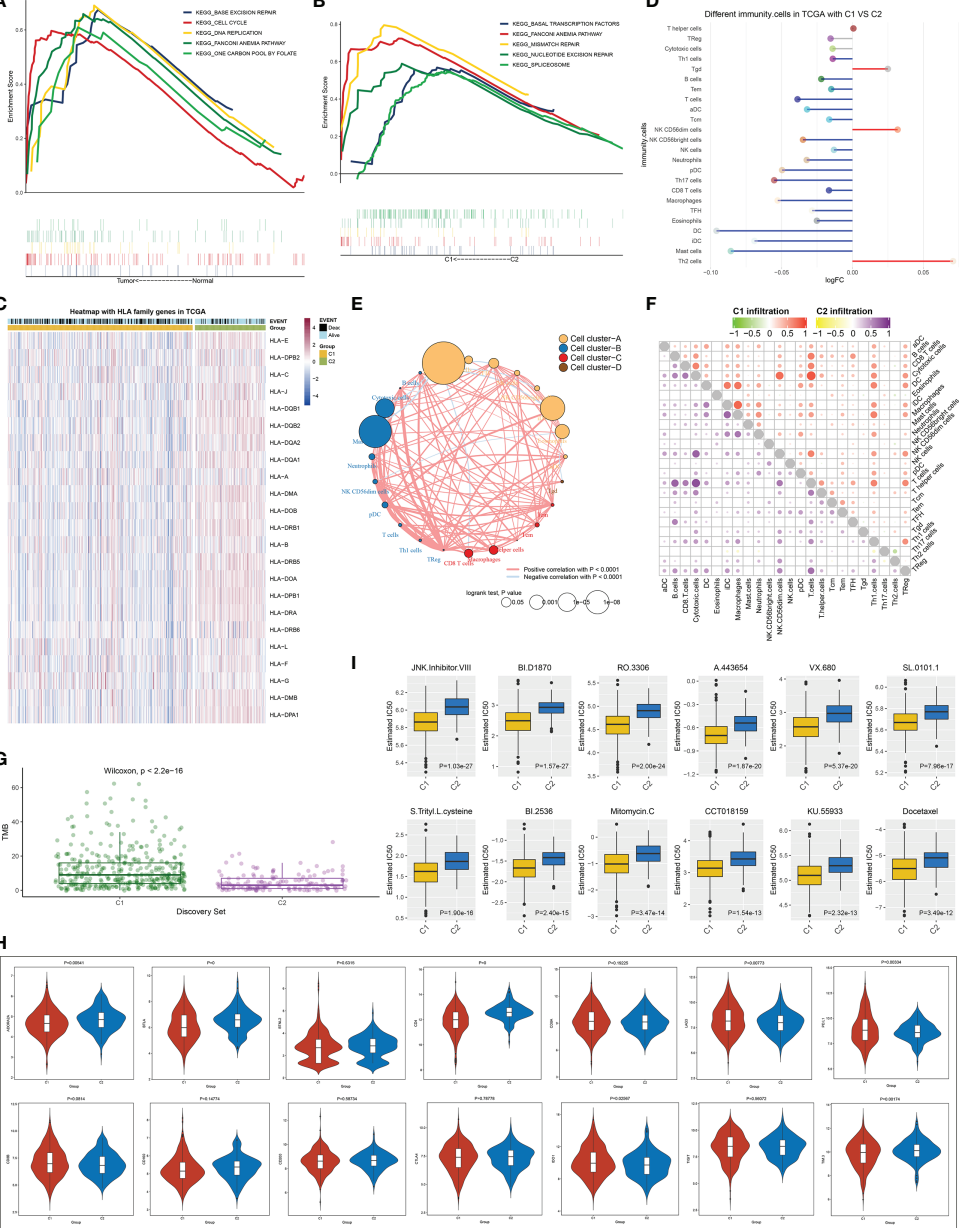
Signaling pathways, antitumor immunity and drug sensitivity in two immune cell infiltration-based subtypes. **(A)** KEGG pathways with different enrichment between LUAD and normal lung tissues. **(B)** KEGG pathways were distinctly enriched between C1 and C2 subtypes. **(C)** Heatmap of the levels of MHC molecules in two molecular subtypes. **(D)** Differences in the abundance of immune infiltrate between subtypes. **(E)** Cluster analysis of various immune cell types across LUAD. **(F)** Correlation of different immune cells with one another within C1 or C2 subtype. **(G)** Difference in TMB score between subtypes. **(H)** Comparison of the mRNA levels of known immune checkpoints in two subtypes. **(I)** Estimated IC50 values of small molecular compounds in two subtypes. KEGG, Kyoto Encyclopedia of Genes and Genomes; LUAD, lung adenocarcinoma; TMB, tumor mutational burden.

GSEA demonstrated that base excision repair, cell cycle, DNA replication, Fanconi anemia pathway, and one carbon pool of folate exhibited a prominent activation in TCGA-LUAD than in normal lung tissues (**Figure 3A**). In both TCGA-LUAD and GSE72094 datasets, in comparison to the C2 subtype, higher activity of basal transcription factors, Fanconi anemia pathway, mismatch repair, nucleotide excision repair, and spliceosome was observed in the C1 subtype (**Figure 3B** and **Supplementary Figure 1**). MHC molecules displayed different expressions between two subtypes across TCGA-LUAD samples (**Figure 3C**). Moreover, we compared the difference in the abundance of immune cells between the two subtypes. Overall, immune cell infiltrations were lower in C1, but with higher infiltration levels of Tgd, NK CD56dim cells, and Th2 cells in C2 (**Figure 3D**; and **Supplementary Table 2**). **Figure 3E** depicts the notable interactions between distinct immune cell types and their prognostic implications across TCGA-LUAD. Afterward, we separately analyzed the correlations between immune cell types in each subtype. As a result, prominent relationships between immune cell types were observed in both C1 and C2 subtypes (**Figure 3F**). TMB may infer the survival benefit of LUAD patients with ICB treatment (27). A higher TMB score was observed in the C1 subtype, which was predictive of clinical benefit with ICB (**Figure 3G**). Immune checkpoints ADORA2A, BTLA, CD4, and TIM3 exhibited higher activity in C2, with lower activity of LAG3, PD-L1, and IDO1 in C1 (**Figure 3H**), indicating the prominent differences in immune checkpoints between subtypes. Moreover, patients in the C1 subtype were more likely to respond to JNK inhibitor VIII, BI-D1870, RO-3306, A-443654, VX-680, SL-0101-1, *S*-trityl-l-cysteine, BI-2536, itomycin C, CCT018159, KU-55933, and docetaxel (**Figure 3I**).

### DNA methylation and genetic mutation of module genes across lung adenocarcinoma

**Figure 4:**
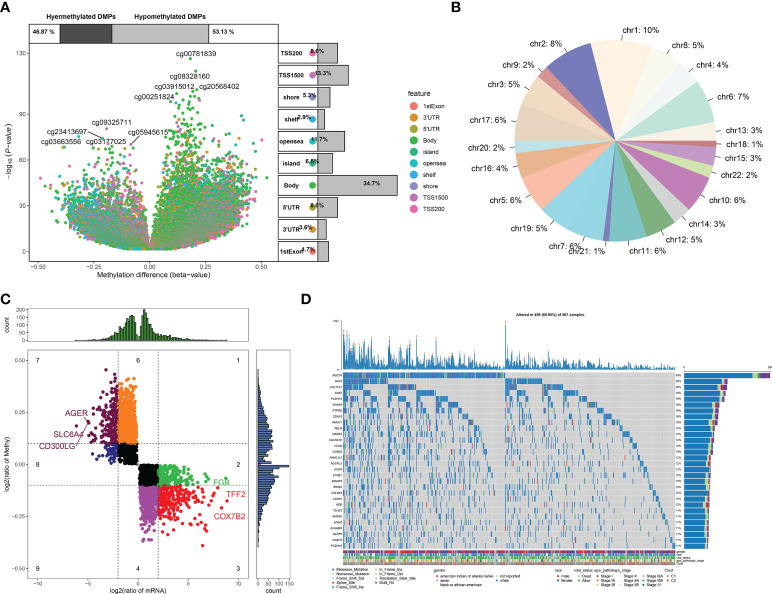
DNA methylation and genetic mutation of module genes across LUAD. **(A)** Differentially methylated sites between LUAD and normal lung tissues. **(B)** Distribution of differentially methylated sites on the chromosomes. **(C)** DEGs that were potentially influenced by DNA methylation. **(D)** Landscape of the top 30 mutated module genes across LUAD. LUAD, lung adenocarcinoma; DEGs, differentially expressed genes.

In TCGA-LUAD dataset, 148,187 differentially methylated sites were determined between LUAD and normal lung tissues (**Figure 4A**). **Figure 4B** visualizes the distribution of differentially methylated sites on the chromosomes. Among differentially methylated genes, 3,340 were module genes whose expression was altered by methylation (**Figure 4C**). Additionally, the genetic mutation was evaluated. Among 567 LUAD samples, 537 (94.71%) occurred genetic mutation, indicating the widespread mutation (**Figure 4D**). According to the mutation frequency, we depicted the top 30 module genes across LUAD samples, including MUC16, NAV3, COL11A1, ANK2, PCDH15, DNAH9, PTPRD, CDH10, HMCN1, RELN, MXRA5, CACNA1E, VCAN, CSMD2, PKHD1L1, ADGRL3, ASPM, SYNE1, BRINP3, RIMS2, COL6A3, LAMA2, NEB, TSHZ3, AHNAK, MYH2, ADGRB3, NLRP3, DNAH3, and PCDH10.

### Identification of key module genes in lung adenocarcinoma

**Figure 5:**
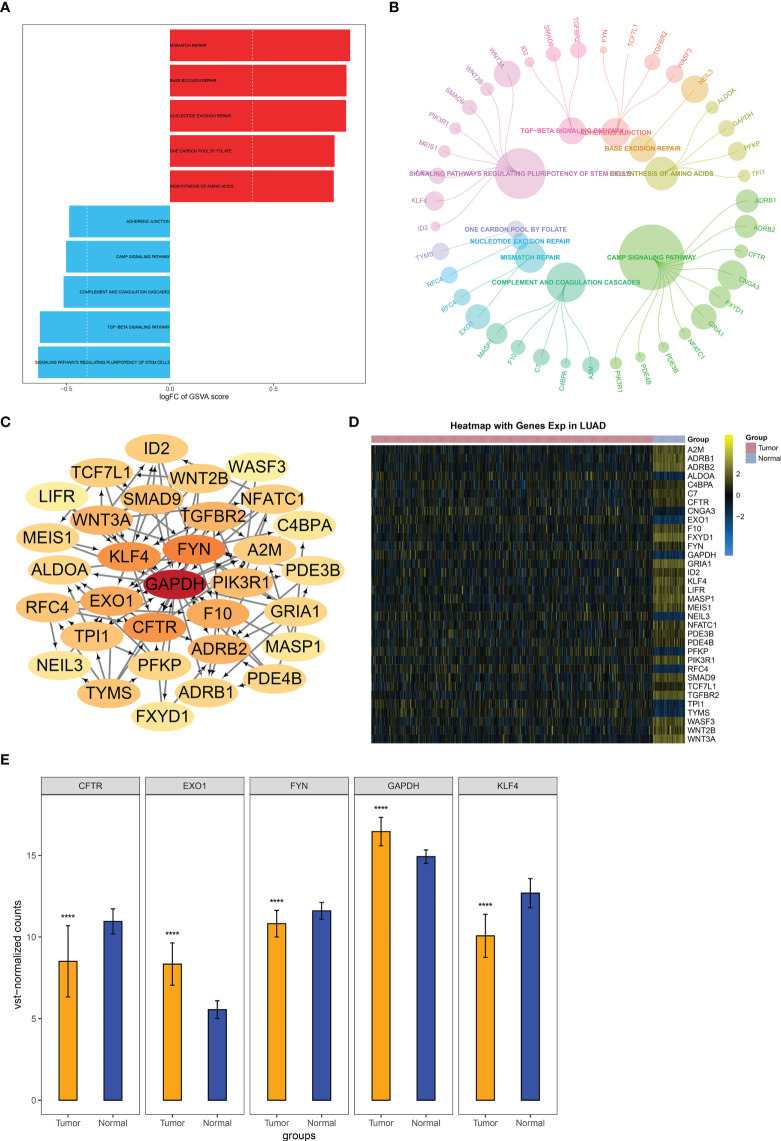
Identification of key module genes in LUAD. **(A)** GSVA of the major pathways enriched by genes in two key modules MEturquoise and MEblue. **(B)** Module genes are involved in the major pathways. **(C)** Selection of key module genes *via* PPI network. **(D)** Heatmap of the expression and methylation of genes from the PPI network in LUAD and normal lung tissues. **(E)** Expression of key module genes in LUAD and controls. ****p < 0.0001. LUAD, lung adenocarcinoma; GSVA, gene set variation analysis; PPI, protein–protein interaction.

Among methylated genes in two key modules MEturquoise and MEblue, 945 were significantly correlated to LUAD cases’ OS. GSVA revealed the top five upregulated pathways (mismatch repair, base excision repair, nucleotide excision repair, nucleotide excision repair, and one carbon pool by folate) and top five downregulated pathways (adherens junction, cAMP signaling pathway, complement, and coagulation cascades, TGF-beta signaling pathway and signaling pathway regulating pluripotency of stem cells) (**Figure 5A**), which were linked to methylated genes (**Figure 5B**). The PPI network showed their interactions (**Figure 5C**). The expression of the above genes between LUAD and controls is depicted in **Figure 5D**. The five genes with the highest connectivity were defined as key module genes, of which GAPDH and EXO1 were upregulated in LUAD, while FYN, CFTR, and KLF4 were downregulated (**Figure 5E**).

### Definition of a scoring system based on key module genes for lung adenocarcinoma prognosis

**Figure 6:**
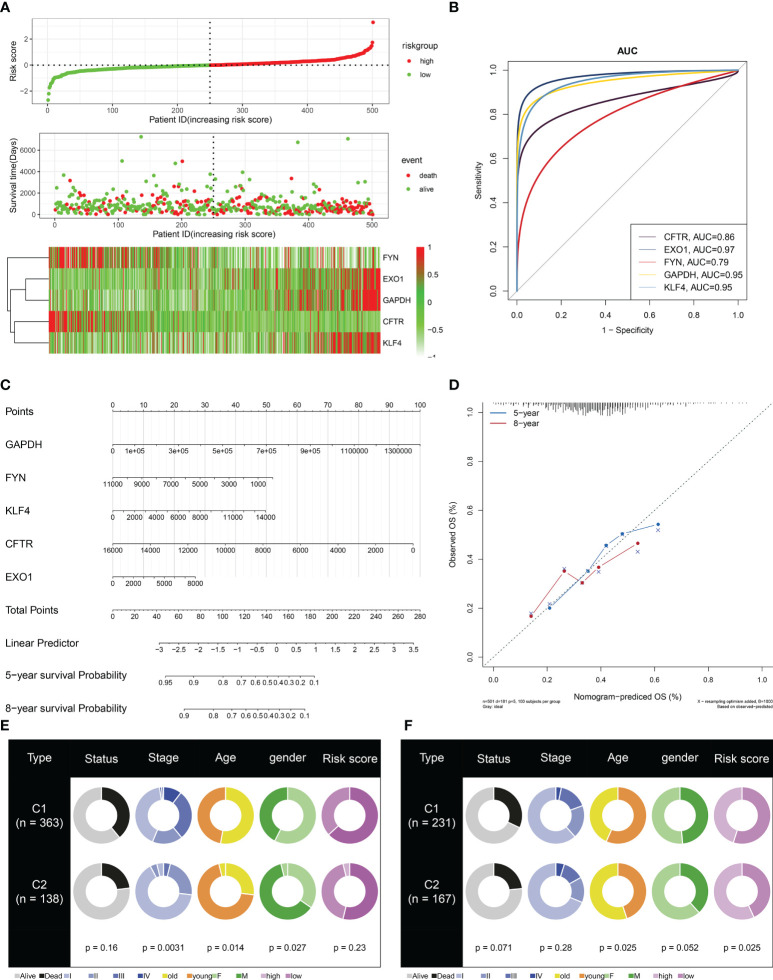
Definition of a scoring system based on key module genes for LUAD prognosis. **(A)** Distribution of risk score, survival state, and mRNA levels of key module genes. **(B)** ROCs for evaluation of the prediction capacity of each key module gene in LUAD prognosis. **(C)** The key module gene-based nomogram for inferring LUAD cases’ OS time. **(D)** Agreement in 5-year and 8-year OS outcomes between the actual data and the nomogram estimation. **(E, F)** Comparison of clinicopathological features and risk score between C1 and C2 subtypes in TCGA-LUAD and GSE72094 datasets. LUAD, lung adenocarcinoma ROCs, receiver operating characteristics; OS, overall survival.

To facilitate the clinical application, the current study established a multivariate Cox regression model of key module genes, and the risk score of each LUAD case was computed. All cases were classified as high- and low-risk groups with the median value (**Figure 6A**). More death cases were observed in the high-risk group. All key module genes had >0.7 AUCs, demonstrating their excellent efficacy in inferring LUAD prognosis (**Figure 6B**). Moreover, a key module gene-based nomogram was established for inferring LUAD cases’ 5- and 8-year OS outcomes (**Figure 6C**). Calibration curves demonstrated that the nomogram performed well in contrast with the ideal model (**Figure 6D**). We also analyzed the differences in clinicopathological features and risk score between C1 and C2 subtypes. In both TCGA-LUAD and GSE72094 datasets, the proportion of stage I–II in C2 was higher than that in C1, indicating better prognostic outcomes for patients in the C2 subtype (**Figures 6E, F**). However, no notable difference in risk score was observed between C1 and C2 subtypes.

### EXO1 correlates to antitumor immunity and is essential for growth of lung adenocarcinoma cells

**Figure 7:**
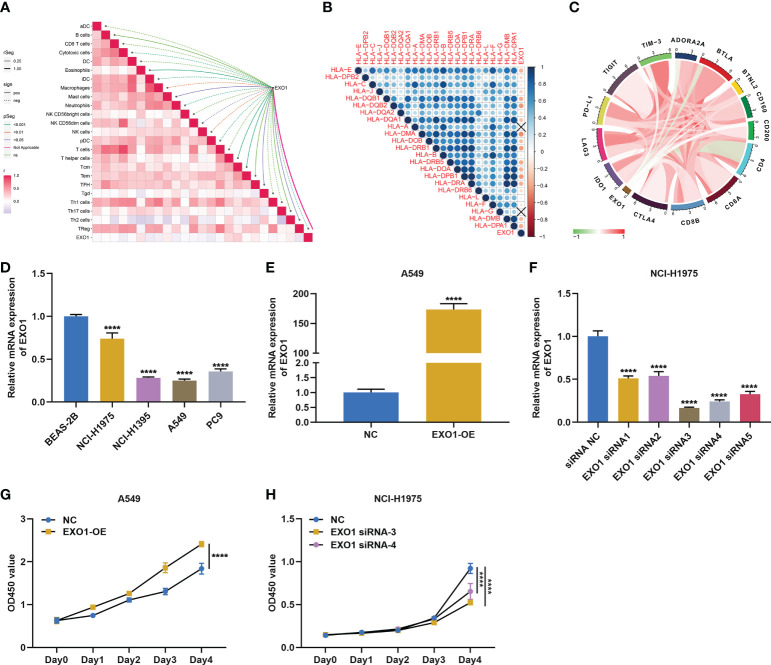
EXO1 correlates to antitumor immunity and is essential for growth of LUAD cells. **(A–C)** Associations of EXO1 mRNA level with **(A)** immune infiltrates, **(B)** MHC molecules, and **(C)** immune checkpoints in LUAD. **(D)** EXO1 mRNA level in human lung normal epithelial and LUAD cells. **(E)** EXO1 mRNA level in A549 cells overexpressing EXO1 (EXO1-OE). **(F)** EXO1 mRNA level in NCI-H1975 cells with EXO1 knockdown (EXO1-siRNA). **(G, H)** CCK-8 for quantifying optical density (OD) to evaluate proliferation of A549 cells with EXO1-OE and NCI-H1975 cells with EXO1-siRNA. ****p < 0.0001. LUAD, lung adenocarcinoma.

Among key module genes, we further focused on EXO1. As illustrated in **Figure 7A**, EXO1 was notably linked to multiple immune cell types. In addition, it prominently correlated to most MHC molecules (**Figure 7B**) and immune checkpoints such as PD-L1 (**Figure 7C**). The above evidence indicated that EXO1 correlated to antitumor immunity in LUAD. To characterize the function of EXO1 in LUAD cells, we first measured EXO1 mRNA level in human lung normal epithelial cell line (BEAS-2B) and LUAD cell lines (NCI-H1975, NCI-H1395, A549, and PC9). Among LUAD cell lines, EXO1 exhibited the highest level in NCI-H1975 and the lowest in A549 (**Figure 7D**). Thus, A549 cells were utilized to overexpress EXO1, with NCI-H1975 cells for its knockdown (**Figures 7E, F**). Overexpressed EXO1 dramatically reinforced the growth of A549 cells, with the opposite findings in NCI-H1975 cells with knockout EXO1 (**Figures 7G, H**), indicating that EXO1 might be essential for LUAD growth.

### EXO1 drives migratory and invasive traits of lung adenocarcinoma cells

**Figure 8:**
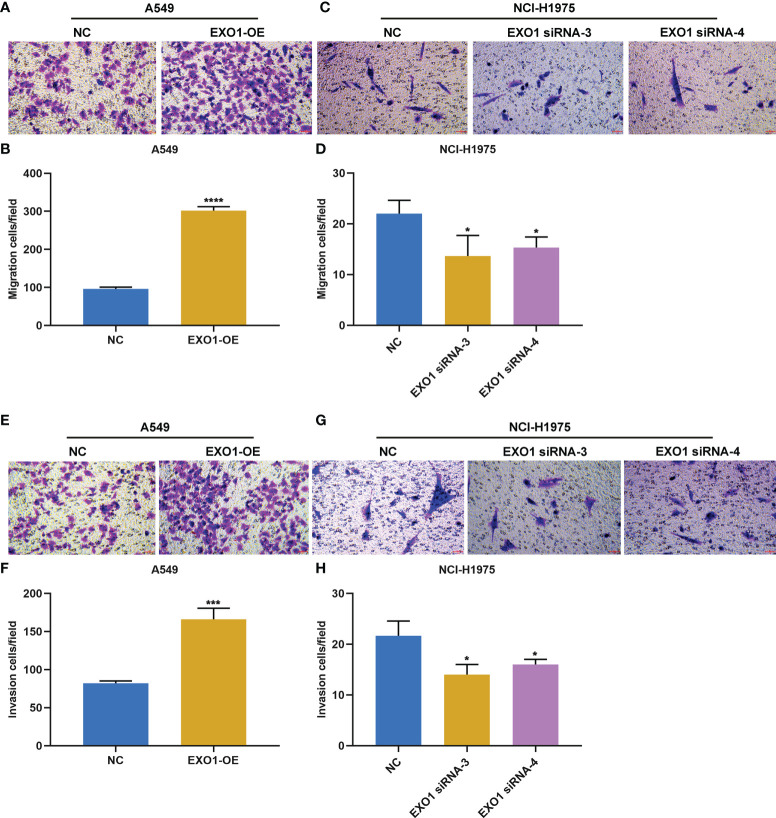
EXO1 drives migratory and invasive traits of LUAD cells. **(A–D)** Representative images of migration assay and number of migratory A549 cells overexpressing EXO1 (EXO1-OE) and NCI-H1975 cells with EXO1 knockdown (EXO1-siRNA). **(E–H)** Representative images of invasion assay and number of invasive A549 cells with EXO1-OE and NCI-H1975 cells with EXO1-siRNA. Bar, 50 μm. NC, negative control; LUAD, lung adenocarcinoma. *p < 0.05; ***p < 0.001; ****p < 0.0001.

The current study further observed whether EXO1 impacted the migration and invasion of LUAD cells. Overexpressed EXO1 notably reinforced the number of migratory A549 cells, while EXO1 knockdown produced the opposite findings (**Figures 8A–D**). In addition, the number of invasive A549 cells was observably heightened by EXO1 upregulation, with opposite findings in NCI-H1975 cells with knockout EXO1 (**Figures 8E–H**). Altogether, EXO1 may motivate migratory and invasive features of LUAD cells.

### EXO1 facilitates PD-L1 and soluble PD-L1 expression in lung adenocarcinoma cells

**Figure 9:**
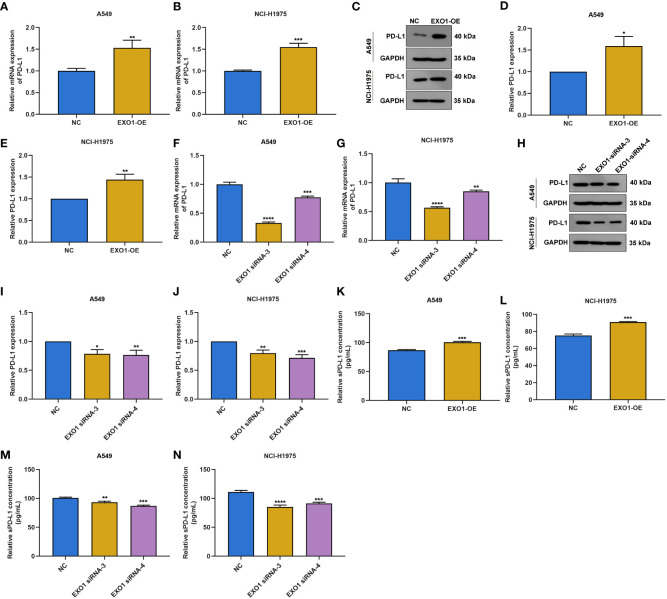
EXO1 facilitates PD-L1 and sPD-L1 expression in LUAD cells. **(A–E)** RT-qPCR and Western blotting of PD-L1 expression in A549 and NCI-H1975 cells with EXO1 overexpression. **(F–J)** RT-qPCR and Western blotting of PD-L1 expression in A549 and NCI-H1975 cells when EXO1 was knocked out. **(K–N)** ELISA for sPD-L1 levels in A549 and NCI-H1975 cells with EXO1 overexpression or knockdown. *p < 0.05; **p < 0.01; ***p < 0.001; ****p < 0.0001. LUAD, lung adenocarcinoma.

Next, we observed whether EXO1 affected PD-L1 expression in LUAD. The results showed that overexpressed EXO1 enhanced PD-L1 expression in A549 and NCI-H1975 cells according to RT-qPCR and Western blotting (**Figures 9A–E**). Oppositely, EXO1 knockdown lowered PD-L1 expression in two LUAD cells (**Figures 9F–J**). In addition, sPD-L1 expression was examined. As a result, EXO1 overexpression elevated sPD-L1 levels in LUAD cells (**Figures 9K, L**), with opposite results when EXO1 was knocked out (**Figures 9M, N**).

## Discussion

Integrating distinct independent datasets to observe the shared key traits of LUAD has become a preferred strategy (28). Due to the bias of individual experiments, it is of significance to seek findings supported by several lines of evidence to boost reliability. Clinical benefits from ICB have been investigated in a minority of LUAD patients (29). Thus, the definition of the appropriate subtypes of patients who can clinically respond to ICB is warranted. The efficacy of immunotherapy is greatly affected by the complex TME especially immune infiltrates. For instance, CD8+ T cells, regulatory T cells, and follicular B cells mediates LUAD progression (29). Thus, it is crucially needed for an in-depth comprehension of how mechanisms orchestrate the tumor immune microenvironment landscape heterogeneity. The present study adopted five LUAD datasets (GSE32863, GSE43458, GSE75037, TCGA-LUAD, and GSE72094) to characterize the immune infiltration landscape across LUAD and unveil relevant epigenetic and transcriptomic mechanisms. Eight immune cell types were notably linked with LUAD prognosis, of which B cells, eosinophils, iDCs, mast cells, T cells, Tem, and Tfh cells acted as risk factors, with Th2 cells as a protective factor. Genetic, epigenetic, cellular, and microenvironmental traits may impact cellular programs and result in diverse pathogeneses of LUAD (30). Here, two immune infiltration-based subtypes were proposed in LUAD, with disparate OS outcomes.

The immunosuppressive microenvironment is essential for the capacity of tumors to avoid detection as well as elimination *via* the immune system (31). To survive under immune pressure, LUAD develops a few mechanisms to evade immune surveillance, primarily *via* expressing immune checkpoints. For instance, PD-L1 expression on tumor-associated macrophages or tumor cells correlates to prolonged survival with adjuvant chemotherapy (32). Here, immune checkpoints ADORA2A, BTLA, CD4, and TIM3 displayed higher levels in C2, with lower levels of LAG3, PD-L1, and IDO1 in C1. TMB acts as an independent marker of beneficial clinical outcomes for LUAD and is in relation to aggressive histological subtypes (33). Additionally, strong evidence has demonstrated the predictive potential of TMB for inferring LUAD cases that have the greatest possibility of responding to ICB (34). A higher TMB score was observed in C1, demonstrating that this subgroup might be responsive to ICB.

Oncogenic mutations are usually regarded as driving factors of tumorigenesis *via* tumor cell-intrinsic mechanisms (35). LUAD exhibits the traits of a high mutational rate of driver genes. Thus, comprehensive genomic testing has become the standard of care in the management of advanced or metastatic LUAD, which enables to determine common or uncommon actionable genomic alterations that influence treatment decisions. Among all module genes, the following genes exhibited the highest mutation frequencies: MUC16, NAV3, COL11A1, ANK2, PCDH15, DNAH9, PTPRD, CDH10, HMCN1, RELN, MXRA5, CACNA1E, VCAN, CSMD2, PKHD1L1, ADGRL3, ASPM, SYNE1, BRINP3, RIMS2, COL6A3, LAMA2, NEB, TSHZ3, AHNAK, MYH2, ADGRB3, NLRP3, DNAH3, and PCDH10. The National Comprehensive Cancer Network guidelines have recommended testing specific molecular as well as immune markers in patients with advanced/metastatic LUAD, thus evaluating applicability for targeted therapy or immunotherapy (36). Stage II to III LUAD patients receive adjuvant chemotherapy to target the premetastatic niche that persists following curative resection (32). Here, the C1 subtype more possibly responded to JNK inhibitor VIII, BI-D1870, RO-3306, A-443654, VX-680, SL-0101-1, *S*-trityl-l-cysteine, BI-2536, mitomycin C, CCT018159, KU-55933, and docetaxel. Previous research has demonstrated the anti-LUAD properties of the above compounds. For instance, BI-D1870 can mitigate tumor growth (37) and potentiate cisplatin activity in LUAD cells (38).

Systematically describing the interactions between immune infiltrate-based subtypes, LUAD and DEGs provided us more insights into the regulatory mechanisms of immunity in LUAD. Moreover, the present study proposed a nomogram on the basis of key module genes with DNA methylation for LUAD. This scoring tool may accurately estimate LUAD cases’ prognosis. EXO1 encodes a protein with 5′ to 3′ exonuclease activity and an RNase H activity, which has an exonuclease domain and a structure-specific endonuclease domain. Consistent with previous research, EXO1 exhibited upregulation in LUAD and was in relation to survival and immune infiltrates within the TME (39). In a Taiwan cohort, it was found that the A allele of Exo1 K589E correlated to an increased risk of lung cancer (40). Additionally, EXO1 Glu589Lys polymorphism and its surrounding regions were potential genetic susceptibility markers of lung cancer in a Chinese population (41). LUAD progresses from tumors that preserve alveolar structure to ones that remodel and eliminate lung structure, reflecting aggressive rather than *in situ* growth properties (42). The current data indicated the contributions of EXO1 to migratory and invasive traits of LUAD cells. In addition, EXO1 triggered the upregulation of PD-L1 and sPD-L1 in LUAD, thus potentially facilitating immune escape. Potential limitations of the present study will be acknowledged. First, due to the retrospective datasets we included, more prospective LUAD cohorts are warranted to verify the conclusion. Second, we only evaluated the reliability of the scoring system in LUAD. More clinicopathological parameters may be integrated into our predictive signature to improve accuracy.

## Conclusion

The current study offered in-depth insights into the multi-dimensional characterization of the immune infiltrate landscape on clinical outcomes and therapeutic response as well as relevant epigenetic and transcriptomic mechanisms in LUAD. The prognostic signature might suggest potential clinical translation in inferring patients’ survival. The function of a key module gene EXO1 in the malignant progression of LUAD was confirmed *via in vitro* assays.

## Data availability statement

The datasets presented in this study can be found in online repositories. The names of the repository/repositories and accession number(s) can be found in the article/**Supplementary Material**.

## Author contributions

LZ, BJ, ZL, JL and QW designed the study and contributed to drafting the manuscript. LZ, BJ, ZL, YY and CY performed experiments, and collated and analyzed the data. LZ, BJ, ZL, JL and QW wrote and revised the manuscript. All authors contributed to the article and approved the submitted version.

## Funding

The study was supported by the Project of Nanning Scientific Research and Technology Development Plan (20183040-4), Guangxi Medical and Health Key Discipline Construction Project (Department of Pulmonary and Critical Care Medicine and Clinical Medical Laboratory Center), Project of Qingxiu District of Nanning Scientific Research and Technology Development Plan (2019038).

## Conflict of interest

The authors declare that the research was conducted in the absence of any commercial or financial relationships that could be construed as a potential conflict of interest.

## Publisher’s note

All claims expressed in this article are solely those of the authors and do not necessarily represent those of their affiliated organizations, or those of the publisher, the editors and the reviewers. Any product that may be evaluated in this article, or claim that may be made by its manufacturer, is not guaranteed or endorsed by the publisher.

## Abbreviations

LUAD, lung adenocarcinoma; ICB, immune checkpoint blockade; TMB, tumor mutational burden; GEO, Gene Expression Omnibus; DEG, differentially expressed gene; WGCNA, weighted gene correlation network analysis; MEs, module eigengenes; ssGSEA, sample gene set enrichment analysis; OS, overall survival; GO, Gene Ontology; KEGG, Kyoto Encyclopedia of Genes and Genomes; BPs, biological processes; CCs, cellular components; MFs, molecular functions; GSEA, gene set enrichment analysis; GSVA, gene set variation analysis; PPI, protein–protein interaction; NC, negative control; siRNAs, short interfering RNAs; CCK-8, Cell Counting Kit-8; OD, optical density; ROC, receiver operating characteristic; AUC, area under the curve.
