# Supplementary figures and images for: Immune infiltration landscape on prognosis and therapeutic response and relevant epigenetic and transcriptomic mechanisms in lung adenocarcinoma
Source: Front Immunol. 2022 Oct 6;13:983570. doi: 10.3389/fimmu.2022.983570 (PMC9582346; doi:10.3389/fimmu.2022.983570)

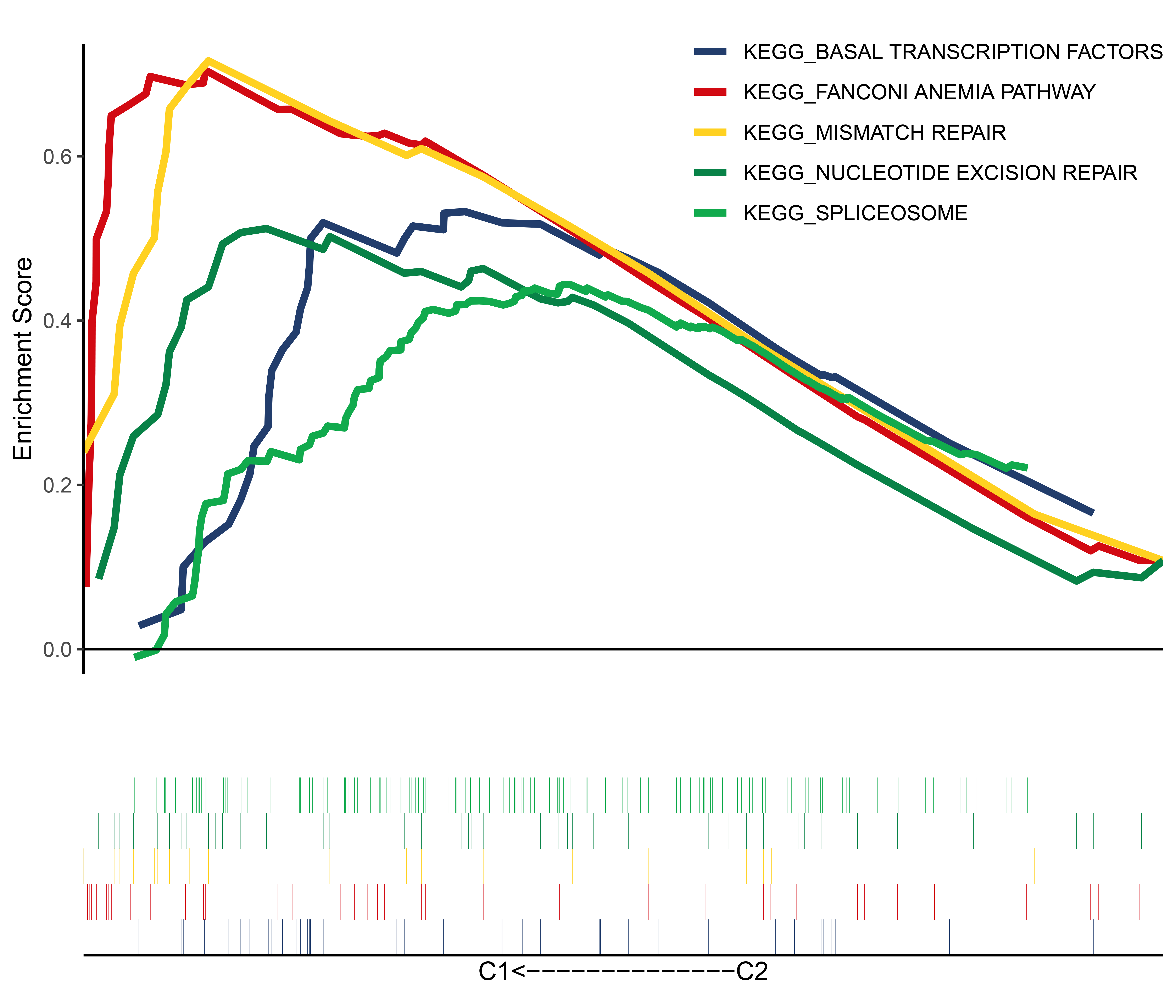

Supplement: Supplementary file 1 [file Image_1.tif]
